# The Integration and Development of Piano Art and Media Education and Its Influence on the Long-Term Care and Happiness of the Elderly People

**DOI:** 10.3389/fpsyg.2021.593835

**Published:** 2021-02-05

**Authors:** Xuan Chen, Fangwei Huang, Yingfeng Wang

**Affiliations:** ^1^College of Music, Hunan Normal University, Changsha, China; ^2^Dongbang Culture University, Seoul, South Korea; ^3^School of Social Science, The Hong Kong University of Science and Technology, Hong Kong, Hong Kong; ^4^School of Art, Jinggangshan University, Ji'an, China

**Keywords:** new media era, piano art, aging, long-term care, happiness

## Abstract

To analyze the influence of the integration of piano art and media on long-term care of the elderly in the aging society, and to improve the living standard and happiness of the elderly, based on educational psychology, several scales of self-compiled personal information, the Ackerson personality inventory, and the memorial university of Newfoundland happiness scale were introduced for statement, and questionnaire method was adopted for information collection. Then, the mechanism of the integration of piano art and media on the happiness of the elderly was summarized. The results showed that there were significant differences in the happiness of the elderly in terms of monthly average income, economic pressure, health status, and living conditions. The happiness of the elderly with a monthly income of more than 5,000 yuan was significantly higher than that of the elderly with a monthly income of <3,000 yuan (*p* < 0.05). The happiness of the elderly with low economic pressure was significantly higher than that of the elderly with high economic pressure (*p* < 0.05). The happiness of the elderly with good living conditions was significantly higher than that of the elderly with ordinary living conditions (*p* < 0.05). The happiness score and positive experience (PE) of the elderly with learning over 5 years were significantly higher than those with learning <5 years and the non-piano learning group (*p* < 0.05). The total score of happiness in the piano learning group was significantly positively correlated with positive affection (PA) and PE, and negatively correlated with neuroticism and psychosis (*p* < 0.05). It is concluded that the piano art learning process based on educational psychology can improve the subjective well-being of the elderly. In addition, the elderly who have been exposed to piano art for a long time are more active in life. Piano art edification is an important factor affecting the psychological well-being of the elderly. The research has a good guiding significance for improving the happiness of the elderly.

## Introduction

With the development of global science and technology and economy, human civilization and science and technology have entered the era of rapid development. The living standard of human beings is gradually improving. But at the same time, various social problems also emerged (Sutipan et al., 2017). With the increasing living standards of human beings, the accumulation of wealth and the high fertility rate, countries are faced with the problem of aging on the road of rapid development (Leubner and Hinterberger, 2017). According to statistics, in developed countries, Japan has taken the lead in the 1970s into an aging society. According to China's statistical data, China's population survey data in 2010 shows that China is about to enter an aging society (Moeini et al., 2018). Based on the above reasons, the Chinese government started to pay attention to the life of the elderly in the early twenty-first century to improve their material life and enrich their spiritual life. According to Eriksson's theory of stages of personality development, the development of human life into old age is a transition from a sense of perfection to a sense of despair. This period is very critical. If it is well-forwarded, the elderly will enjoy their old age peacefully and have full confidence in life. However, if the transformation is not good, the elderly will feel disappointed and pessimistic in the later stage of old age (Moreno-Gómez et al., 2017).

According to Eriksson's theory, on the road to the aging society, in order to enable the elderly to have a good living condition and psychological state in their later years, the material life and mental state of the elderly have received unprecedented attention from the government and related fields. The government actively invests in the construction of the living security system for the elderly and improves their material security base (Luchesi et al., 2018). Relevant scholars have started a large number of studies on elderly people's happiness experience. With the development of research, a lot of research results have been obtained. According to the report and related research, among the gray-haired, happiness is mainly affected by age, long-term care, location, and environment. These four factors are the main factors that affect the happiness of the elderly. Music art learning under the influence of environment provides a good direction for the study of happiness of the elderly. Long-term care refers to the continuous care and nursing services provided for people with chronic diseases or disabilities, namely functional impairment, over a long period of time. It is mainly to provide comprehensive and professional care, rehabilitation nursing, spiritual consolation, social interaction, and hospice care for the disabled and semi-disabled (Fu et al., 2017). Educational psychology is the study of human learning, the effect of educational intervention, the psychology of teaching, and the social psychology of school organization under the educational context. The emphasis of educational psychology is to apply the theory or research of psychology to education. Many studies have found that music art learning based on educational psychology has a very close relationship with the happiness of the silver haired. In particular, piano learning can not only improve the physical and mental health of the elderly, adjust their living conditions, but also improve their interpersonal communication level (Tran et al., 2018). The research on the physiological and psychological functions of music in human body has never stopped from ancient times to now, from theory to practice. With the development of the times and the change of media means, the research on the influence of the integration of piano art based on educational psychology and media education on the long-term care and happiness of the elderly in the era of new media is of great significance.

To sum up, the improvement of life happiness of the elderly is one of the main objectives of current research, and piano art learning based on educational psychology may promote the happiness of the elderly. Based on this, to study the influence of the integration of piano art and media education on the happiness of the elderly in the new media era, statistical correlation method is adopted to study the relationship between piano art and media education integration development in the new media era and long-term care and happiness of the elderly. Firstly, the integration and development of piano and media in the new media era is introduced. Then, by means of questionnaire survey, an empirical analysis is made on the influence of piano art and media education integration on the long-term care and happiness of the elderly in the new media era. Finally, the mechanism of the integration of piano art and media education in the new media era on the long-term care and happiness of the elderly is introduced. It is hoped that the study can provide a good way to care for the life of the elderly with the help of educational psychology.

## Literation Review

### Foreign Related Research

With the development of society and the improvement of human civilization, the degree of aging in every country in the world is increasing to different degrees. In the whole social system, the team of silver hair is getting larger and larger, which has gradually attracted the attention of the whole society. In addition, with the development of science and technology, the question whether the elderly can keep up with the pace of the development of science and technology has led to more and more researches on the elderly (Biasutti and Mangiacotti, 2019). Abroad, most of the research is on the elderly people's happiness in their later years. Most studies indicate that music learning based on educational psychology has a close relationship with happiness in the elderly. This study leads to the fact that the elderly is learning music-related activities in their later life (Ken-Ichi et al., 2017). Gabrielssonl and Darboe (2017) have found that learning music can induce and process human emotional peak, and there is a close relationship between music elements and strong music experience. Joseph et al. (2018) found that there is a hormone molecule in human body, which can make people's mood become calm, and this hormone molecule can be activated by listening to music. Hill (2017) empirically found that music can bring people a short period of positive emotional experience, reduce people's irritable mood, and make people put down their aggressiveness and vigilance. Willian et al. (2017) found in their research that regular listening to music and participating in various music activities can help the elderly to live a healthier life.

### Domestic Related Research

In China, people began to study the effect of music on the life of the elderly. Ren (2017) found that when learning piano, the elderly could significantly improve their visual and auditory abilities as well as their ability to process and remember materials. Fan et al. (2017) found a positive correlation between participation in learning activities and subjective well-being of the elderly and their subjective well-being. Although the study did not mention the relationship between piano learning and subjective well-being in the elderly, the study confirmed the relationship between learning activities and well-being in the elderly. He et al. (2018) compared piano learning with the elderly who did not participate in piano learning, and found that enjoying music during piano learning can improve the happiness of the elderly. Wu et al. (2019) found through research that elderly people who love learning music have extroverted personality, and learning music has certain influence on personality.

### Comparison and Summary of Related Foreign and Domestic Research

Through the above study, it was found that most of the literatures studied the improvement of subjective well-being of elderly women when they studied in music, as well as the influence of piano learning on the two factors of intelligence and psychology of the elderly. But with the development of society, technology has changed people's way of life. Under the new media era, the research on the influence of piano art and media education integration and development on the long-term care and happiness of the elderly has not been involved. In view of the above, statistical methods are adopted to study the influence of piano art and media education convergence development in the new media era on the long-term care and happiness of the elderly.

## The Integration and Development of Piano and Media in the New Media Era

### Development Characteristics of Piano Art in the New Media Era

The twenty-first century is an era of information, and the forms of media are also undergoing great changes. Information technology has changed the way media is produced, from CDS to digital products. And with the development of the Internet, the earth is like a village. Information exchange is more convenient with the help of network change. All kinds of information can be exchanged online at anytime and anywhere (Yap et al., 2017). The piano is no exception. The world's piano music has changed from the previous non-cooperation and non-communication, and the piano music has different music colors in different places, to now it starts to discuss with each other, learn from each other, and integrate and improve different music styles. For example, the music creation of the famous French impressionist master Debussy is a typical representative of absorbing various artistic aesthetic thoughts and integrating the spirit of eastern and western cultures. In his piano creation, the rich oriental colors such as pattern, melody, rhythm and content make his piano works present different aesthetic feelings. At the same time, the creation of Chinese piano works is mainly reflected in the combination of the creation technology of western piano with the music characteristics of China's own nation, the use of folk songs and folk instrumental music including the features of opera and other national music, and the use of the harmony and tone of national music. For example, the first winning piano work in China, the shepherd boy piccolo by Mr. He luting is the first winning piano work in China (Lamont and Ranaweera, 2019). Therefore, it shows that in the era of new media, the communication and development of world piano culture will face greater challenges.

News media pay great attention to audience feedback on their programs. At present, the construction and popularization of mobile network platform make this function of news media have unprecedented improvement. Two people far away from each other can communicate and communicate with each other through radio, television, audio and video products. They can have easy access to elegant piano music anytime and anywhere. But this mode of transmission will not change the alienation of piano art. Elegance doesn't mean “highbrow.” In the new media era, the interaction between the media and the audience is stronger than before, which shortens the distance between the public and the piano art. People can “touch” the piano art through various channels to increase “face-to-face” interaction with piano players. Piano teachers and students can also communicate and interact with each other timely through online teaching, which increases the opportunities for the elderly to contact with piano art. Piano educators across the country can exchange experience through remote online teaching, and the piano culture can also be spread to remote villages that have been illegally contacted before, which is expected to realize the systematic and comprehensive development and modernization of piano art. At present, the development of piano art must conform to the characteristics of modern communication (Mazaheryazdi et al., 2018).

An important feature of the development of piano art in the new media era is the integration of multimedia technology. Multimedia refers to text, graphics, images, video, voice and other media information, with interactive, collaborative, real-time, integrated media technology. The application of multimedia electronic technology can greatly enrich and expand the traditional piano performance and teaching. Multimedia keyboard instruments are mature with the development of multimedia music technology, such as two-line piano. Due to the integration of multimedia electronic technology, it has a powerful function and integration ability. Compared with traditional piano, it has better mobility and practicability, which is favored by the industry (Paquette et al., 2018). Digital piano teaching as a new teaching method, is getting more and more foreign attention. Rich multimedia digital piano classroom performance and rich teaching content, as well as seamless connection with multimedia audio technology, can quickly and effectively train and improve the comprehensive use ability of keyboard for the elderly (Zhang et al., 2018).

### The Relationship Between the Development of Piano Art and the Long-Term Care of the Elderly Under the Background of New Media Era

With the rapid development of new media, the lifestyle of the elderly has undergone great changes. Aging is an irresistible law of nature. Long-term care for the elderly can improve their psychological state and greatly improve their happiness in their later years. Long-term care for the elderly mainly includes physical care and mental care. Physical care is divided into family care, centralized institutional care and community care. The study found that a large proportion of elderly people's better happiness experience in their later years comes from the care of family and children. The second is centralized institutional care, and the last is community care. Mental care improves the lives of the elderly psychologically. Changes in media products have also changed care for the elderly. Piano training in old age used to be one-on-one tutoring by teachers, but now it is online. The elderly needs less company, which makes their happiness experience greatly discounted. At present, the development model of long-term care for the elderly in China is to advocate self-help and mutual support for the elderly, based on family support, home care for the elderly as the main, supplemented by institutional care, and the state, and better laws, regulations and policy protection are given by the government. With the development of new media, long-term piano art is also a kind of mental care for the elderly. The study of art can improve the mental state of the elderly.

## The Integration and Development of Piano Art and Media Education in the New Media Era: An Empirical Analysis on the Long-term Care and Happiness of the Elderly in the Aging Society

### The Process of Questionnaire Survey

There are three research tools used in this study: self-compiled personal information, the Ackerson personality inventory, and the memorial university of Newfoundland happiness scale.

Firstly, Self–compiled personal information questionnaire. According to the contents of demographic variables, a set of questionnaires about the factors influencing happiness predicted was designed by demographic variables, which covered personal basic situation and music learning.

Secondly, the Eysenck personality questionnaire was developed by the British psychologist. Subsequently, Gong et al. (2014) Chinese scholars, revised it based on China's situation and compiled a set of personality questionnaire suitable for adults (Bowden et al., 2018; Liu et al., 2018). The questionnaire consists of four subscales, including introversion and extroversion (E), neuroticism (N), psychosis (P), and cover-up (L). Among them, cover-up (L) is an indicator to measure whether lying or not, which has little influence on this questionnaire, so it is not included in the analysis.

Thirdly, Memorial university of Newfoundland happiness scale (Table 1) was developed by Canadian scholar (Albert et al., 2011) based on the theory of emotional balance, the theory of emotional balance refers to the balance between positive emotions and negative emotions. Positive emotion, as its name implies, is an emotion that positively increases people's happiness, while negative emotion is an emotion that produces negative emotion to reduce people's happiness (Charan et al., 2018). Positive and negative emotions are mutually independent and balanced. The scale has 24 items. There are three grades for each question, namely 0, 1, and 2. The internal consistency reliability of each subscale was good in this study.

**Table 1:** The distribution of the well-being scale at memorial university of Newfoundland.

**Factors**	**Number of entries**	**Score ranges**
Positive affection (PA)	5	0~10
Negative affection (NA)	5	0~10
Positive experience (PE)	7	0~14
Negative experience (NE)	7	0~14

*Total score of subjective well-being=PA-NA+PE-NE, (to get a positive value, usually plus a constant of 24, scoring from 0 to 48)*.

SPSS22.0 was adopted for data analysis in this study, and ANOVA, correlation analysis and multiple regression analysis are performed.

**Figure 1:**
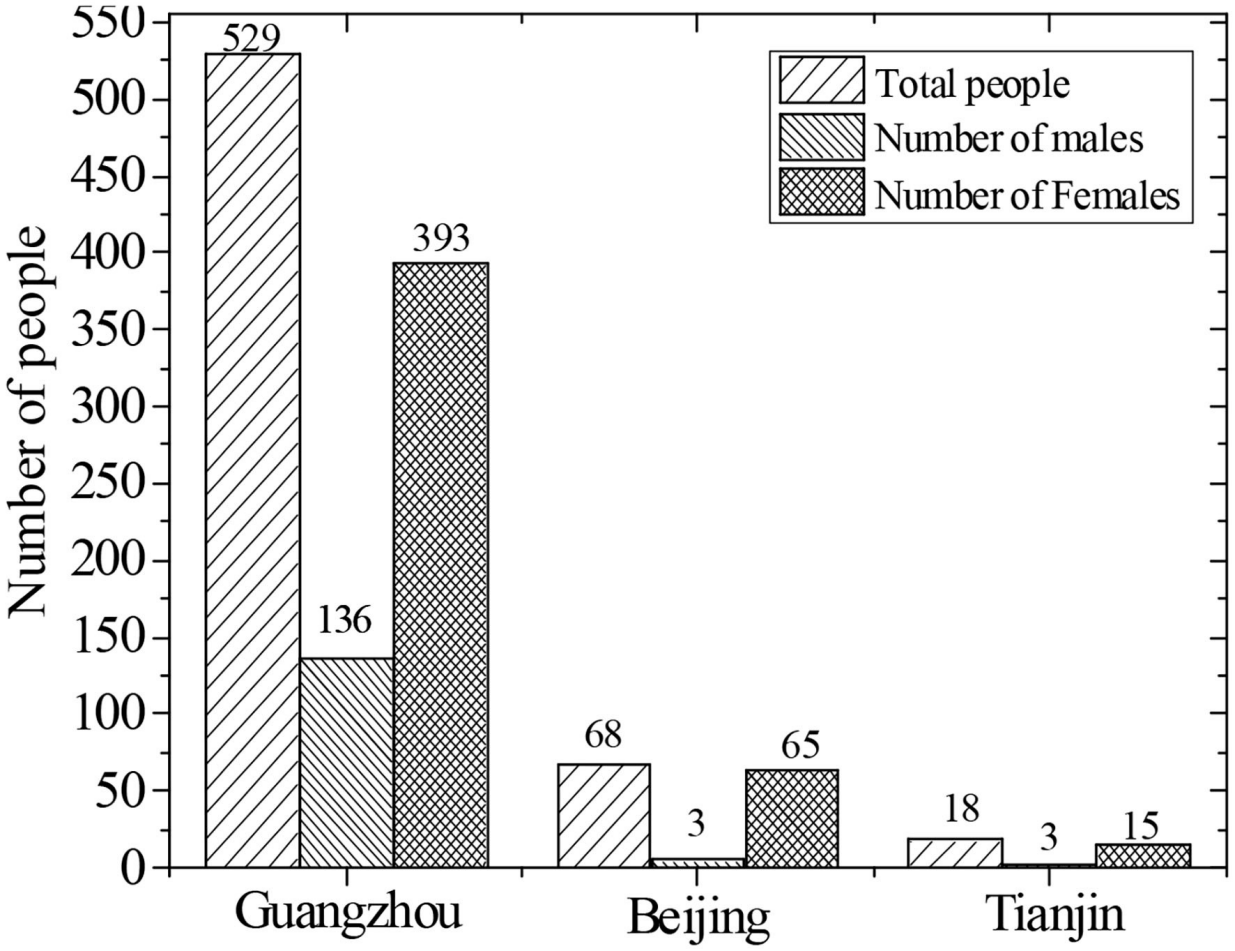
Figure of survey number and gender distribution of the subjects by region.

A random survey method was adopted to study the influence of piano art and media education integration and development on the long-term care and happiness of the elderly in the age of new media. The survey was conducted between January and June 2019. A questionnaire survey was conducted in Guangzhou, Beijing and Tianjin on the elderly who participated in piano learning and the elderly who did not. The purpose and method of this survey, as well as the matters needing attention to fill in the questionnaire, were explained to the elderly in the study unit by the researcher. Let the elderly have a clear understanding of this survey, which can ensure the accuracy of the questionnaire survey better. A total of 1,000 questionnaires were distributed in this survey, and the respondents were all elderly people in good mental condition and mental health. A total of 617 valid questionnaires were collected. The information table of the respondents in the questionnaire survey is shown in Table 2, and the number of recovered parts in the questionnaire is shown in Figure 1.

**Table 2:** Questionnaire survey information table of subjects.

**Questionnaire issuing**	**Region**	**Total number**	**Number of**	**Number of**
**unit**		**of people**	**males**	**females**
Beijing university for the aged	Beijing	68	3	65
Piano study group for the elderly in Tianjin	Tianjin	18	3	15
Shenzhen university for the aged	Guangzhou	367	102	265
Piano amateur competition for the elderly	Guangzhou	162	34	128

**Figure 2:**
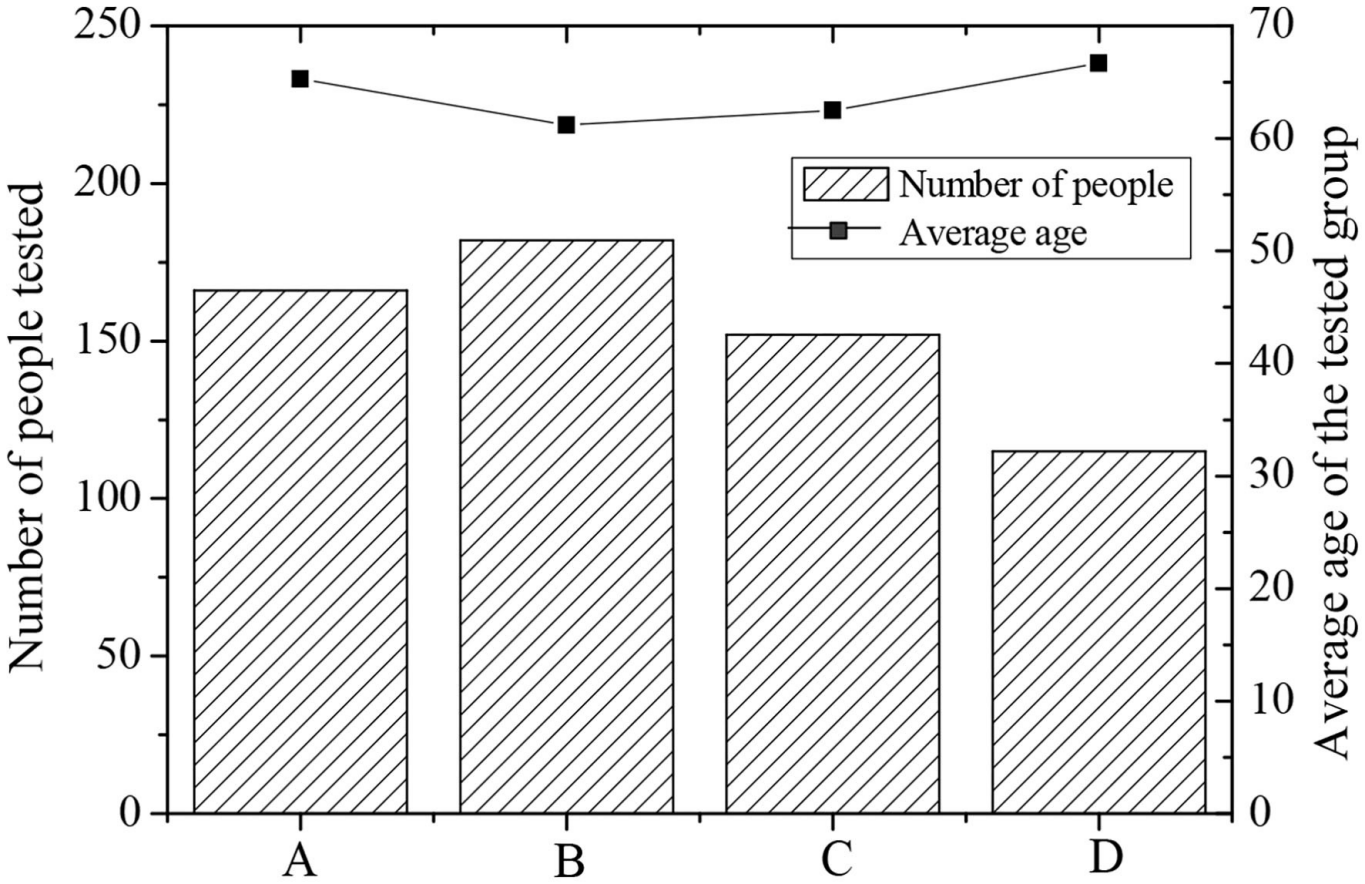
The number of members of each group and the average age distribution (Group A: non-piano study group; Group B: study group <2 years; Group C: study group with 2–5 years; Group D: study group with more than 5 years, Figure 3~ Figure 5 the same).

According to the statistical results collected from the questionnaire survey, the duration of piano learning was divided into four groups. They are the non-piano study group, the study group with <2 years, the study group with 2–5 years, and the study group with more than 5 years, respectively. Figure 2 shows the distribution of the number of members in each group and the average age.

### Analysis of Factors Influencing the Long-Term Care of the Elderly and Subjective Well-Being Under the Background of Piano Art and Media Education Integration and Development in the New Media Era

The long-term care of the elderly needs to be analyzed according to the basic living conditions. In the basic living conditions of the elderly, there is no statistical difference in the influence of marriage status, retirement years and other factors on the subjective well-being of the elderly. However, there are significant differences between monthly average income, economic pressure, health status, living conditions and the well-being of the elderly, as shown in Table 3.

**Table 3:** Analysis of differences in demographic variables.

**A variable factor**		***N***	**Total score**	**Standard deviation**	***F***	***P***
Average monthly income	Less than 3000 yuan	214	34.68	7.76	3.448	<0.05
	3000~5000 yuan	136	35.49	7.69		
	More than 5000 yuan	99	37.10	7.09		
Economic pressure	Little or no	161	37.83	6.84	12.723	<0.01
	General	187	34.27	7.73		
	Greater	101	33.99	7.71		
Health condition	Healthy	169	38.36	7.34	26.431	<0.01
	General	225	34.46	6.56		
	Not very healthy	55	30.96	9.14		
Living condition	Very good	166	38.17	7.35	33.447	<0.01
	General	262	33.78	7.23		

According to Table 3, the happiness of the elderly with a monthly income of more than 5,000 yuan was significantly stronger than that of the elderly with a monthly income of <3,000 yuan (*p* < 0.05). The happiness of the elderly with low economic pressure was significantly higher than that of the elderly with high economic pressure (*p* < 0.05). The happiness of the elderly with good living conditions was significantly higher than that of the elderly with ordinary living conditions (*p* < 0.05). The higher their income, the more ways they were cared for, and the higher their subjective well-being. There was a significant difference between low economic pressure and high economic pressure. With low economic pressure, the more ways of caring, the higher the subjective happiness. The better the living conditions, the stronger the subjective well-being.

### The Overall Evaluation and Comparison of Subjective Well-Being of the Elderly Piano Art and Media Education Integration and Development in the New Media Era

The overall score of subjective well-being and the scores of PA, NA, PE, and NE of the elderly in the four groups investigated are shown in Table 4. It showed that, in terms of overall score, the total score of subjective well-being, positive temperament and positive emotional experience of the elderly who did not participate in piano art study were all below the average score of the scale. The total score of subjective well-being, positive temperament and positive emotional experience of the elderly who participated in piano art study were all above the average score of the scale. The total score of happiness, PE, NA, and NE had obvious difference between groups with different piano learning time. The total score of happiness and PE of the elderly with more than 5 years leaning were significantly higher than those with learning <5 years and the non-piano learning group (*p* < 0.05). NA and NE of the elderly with more than 5 years of learning were significantly lower than that of the those with <5 years of learning and non-piano learning group (*p* < 0.05). There was no significant difference in PA between the elderly with more than 5 years of learning and those with <5 years of learning and those in the non-piano study group (*p* > 0.05).

**Table 4:** The difference between the total happiness score and each factor score of the elderly and the four groups.

**Group**	**Age**	**Total score**	**PA**	**NA**	**PE**	**NE**
Non-piano study group	64.9 ± 8.00	32.79 ± 8.65	5.32 ± 2.54	2.32 ± 2.24	9.06 ± 3.34	3.32 ± 3.14
Study group <2 years	61.5 ± 7.00	34.81 ± 7.91	5.72 ± 2.46	1.94 ± 2.01	10.0 ± 2.96	3.02 ± 2.46
Study group of 2~5 years	62.4 ± 7.14	34.96 ± 7.47	5.54 ± 2.38	1.82 ± 1.97	9.84 ± 3.08	2.54 ± 2.48
Study group more than 5 years	66.5 ± 6.83	37.12 ± 7.11	6.12 ± 2.29	1.65 ± 1.79	10.7 ± 2.69	2.12 ± 2.29
In total	63.5 ± 8.03	34.73 ± 8.02	5.67 ± 2.45	1.94 ± 2.03	9.84 ± 3.05	2.87 ± 2.45
*F*	15.994^**^	6.714^**^	2.001	3.394^**^	7.269^**^	5.014^**^

** *means the difference is very significant (p < 0.01)*.

**Figure 3:**
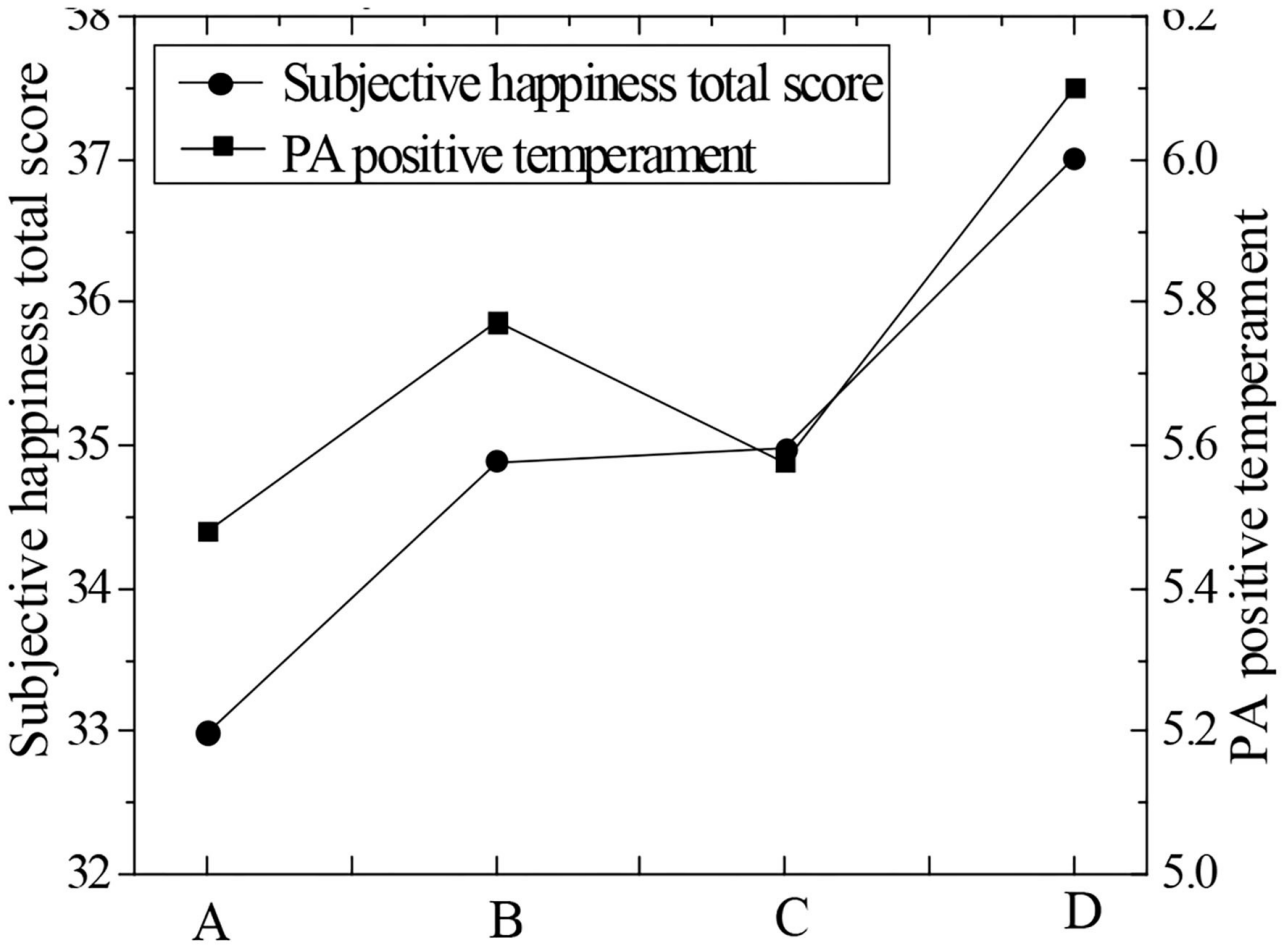
Plot of mean value of total happiness score and PA positive emotion score of the four groups.

**Figure 4:**
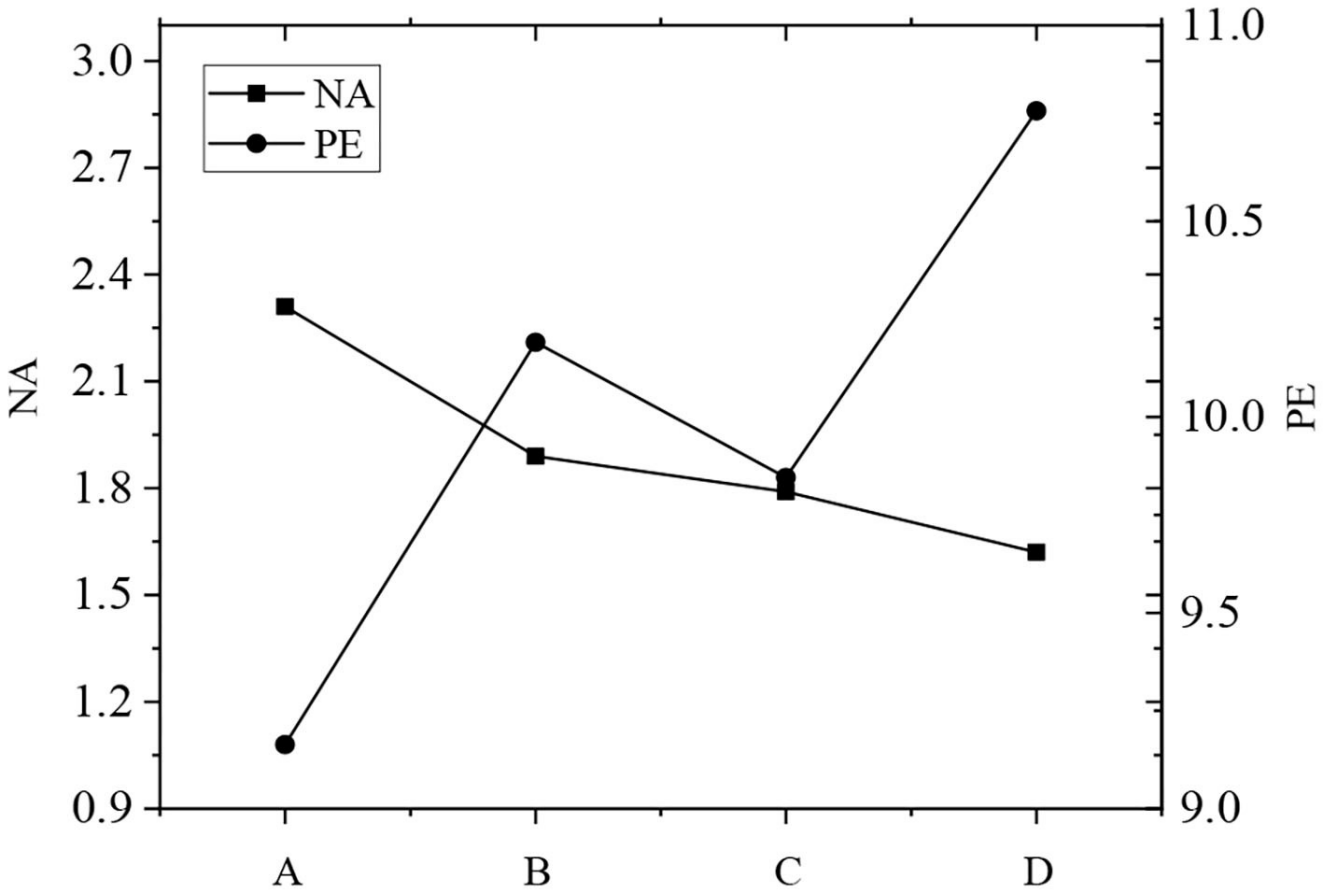
Mean score of positive emotion of NA and PE.

**Figure 5:**
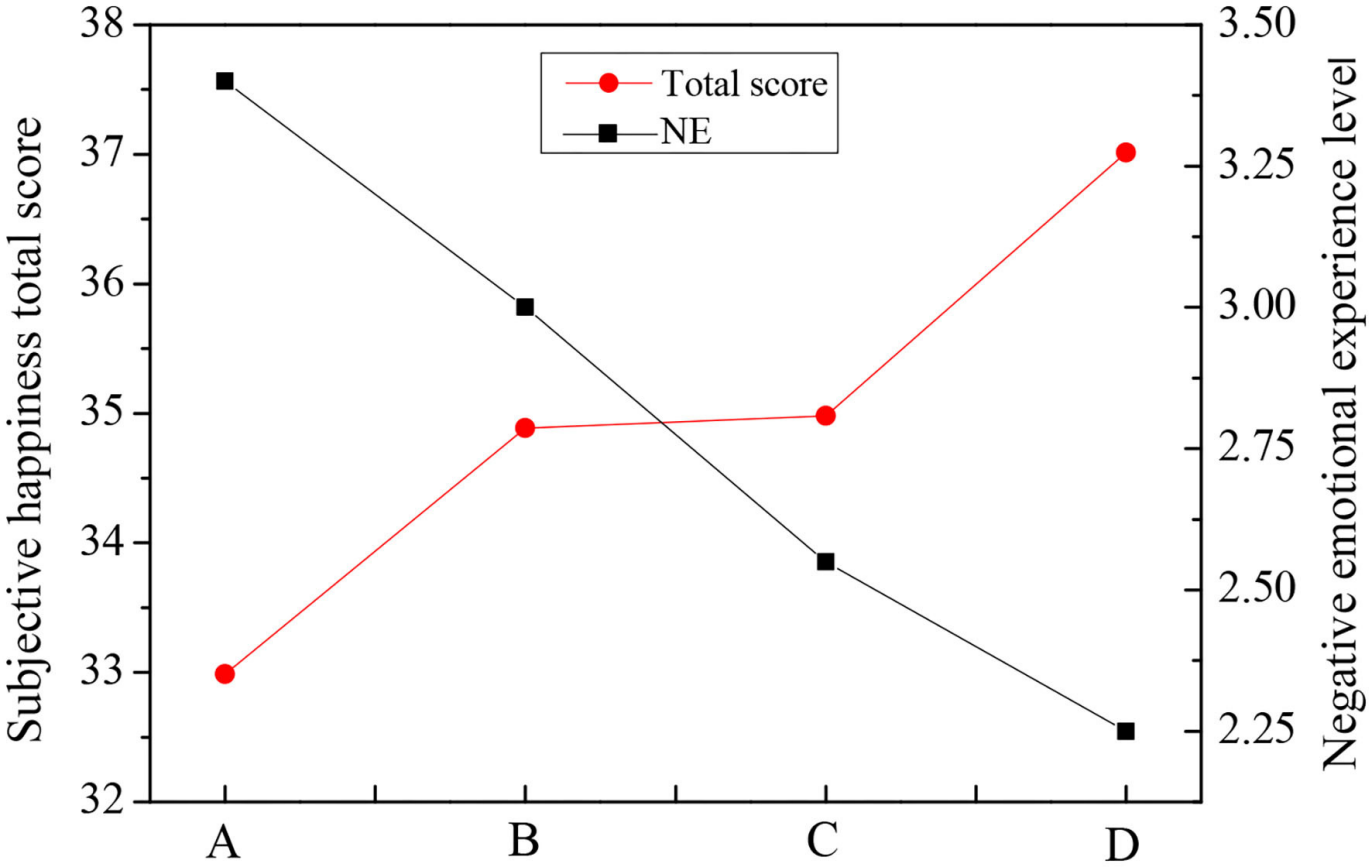
The mean value of total happiness score and NE positive emotion score of the four groups plotted.

It shows in Table 4 that the scores of PA and PE were both greater than the scores of the scale. The scores of NA and NP were both lower than the scores of the scale. Among all the positive factors, the longer the piano learning time, the higher the score. Emotional factor scores of each group were plotted as Figures 3–5. According to Figure 3, the total subjective well-being score and PE score increased with the increase of piano art learning time, and NA decreased with the increase of piano art learning time. NE has been declining as the time spent studying piano increased. From Figures 3–5, it showed that both positive emotion and positive emotional experience increased with the increase of piano learning time, while negative emotion and negative emotional experience decreased with the increase of piano learning time, indicating that piano art learning based on educational psychology can improve the happiness of the elderly. With the development of modern science and technology, in the age of new media, the elderly has more and more access to piano media, so it has a positive effect on the well-being of the elderly.

According to the statistical analysis of the subjective happiness of the elderly in the piano learning group (Table 5), with the learning time of the elderly as the variable, the subjective well-being of the elderly in the piano learning group was taken as the dependent variable, and the variance analysis showed that there were significant differences among the three groups in the acquisition of happiness. Among them, PE and NE factors were significantly different, while PA and NA were not. And multiple comparisons were made (Table 6). Therefore, it also indicated that in the subjective well-being of the elderly, positive emotion and positive emotional experience increased with the increase of piano learning time, while negative emotion and negative emotional experience decreased with the increase of piano learning time.

**Table 5:** The score of four factors between the piano learning groups and the comparison of the differences between the groups.

**Group**	**Sample size**	**Age**	**Total score**	**PA**	**NA**	**PE**	**NE**
Study group <2 years	183	61.5 ± 7.00	34.8 ± 7.91	5.72 ± 2.46	1.94 ± 2.01	10. ± 2.96	3.02 ± 2.46
Study group of 2~5 years	152	62.4 ± 7.14	34.9 ± 7.47	5.54 ± 2.38	1.82 ± 1.97	9.84 ± 3.08	2.54 ± 2.48
Study group more than 5 years	115	66.5 ± 6.83	37.1 ± 7.11	6.12 ± 2.29	1.65 ± 1.79	10.7 ± 2.69	2.12 ± 2.29
In total	450	63.5 ± 8.03	34.7 ± 8.02	5.67 ± 2.45	1.94 ± 2.03	9.84 ± 3.05	2.87 ± 2.45
F			3.579^*^	1.906	1.513	3.414^**^	3.669^**^

* means the difference is significant (p < 0.05);

** *means the difference is very significant (p < 0.01)*.

**Table 6:** Comparison of multiple differences of subjective well-being total score and factor score in piano learning group.

	**Total score**			**PE**			**NE**		
	**B**	**C**	**D**	**B**	**C**	**D**	**B**	**C**	**D**
**B**			^*^			^*^			^**^
**C**			^*^			^*^			

*B: study group <2 years; C: study group with 2–5 years; Group D: study group with more than 5 years*.

* means significant difference (p < 0.05);

** *means the difference is very significant (p < 0.01)*.

**Figure 6:**
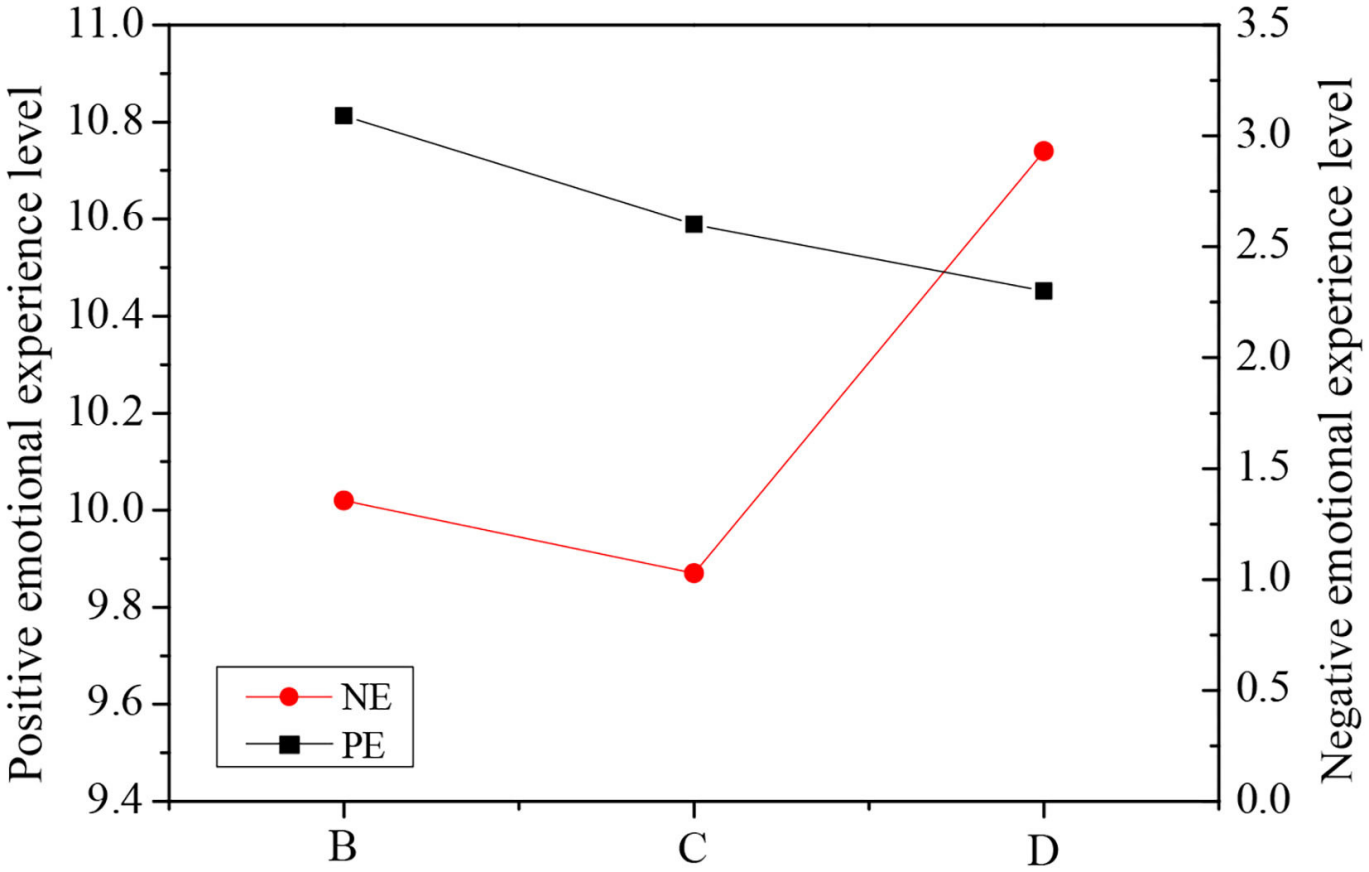
Comparison of PE and NE among the elderly in different time study groups.

According to the variance results, there was no significant difference between PA and NA factors in the piano learning group, so these two factors are not analyzed in the research. Figure 6 shows the comparison of PE and NE between the study groups of the elderly at different times.

**Figure 7:**
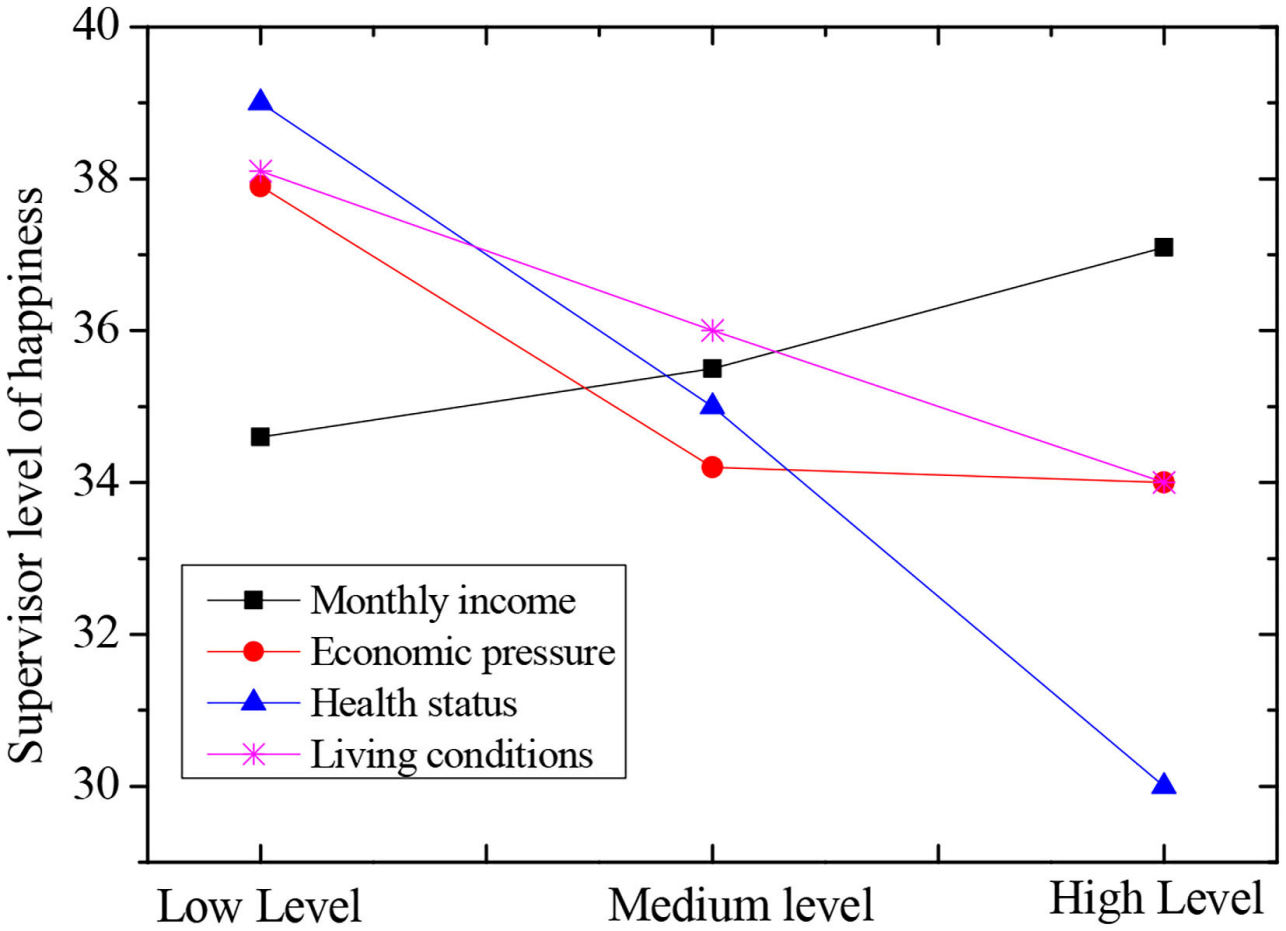
Comprehensive evaluation of different influencing factors and happiness.

With personality as the variable, the subjective well-being of the elderly in the piano learning group was taken as the dependent variable to conduct correlation analysis. There were three factors affecting personality variables, and the correlation analysis between them and the three factors of personality variables was shown in Table 7. The results showed that the total happiness score of the elderly in the piano learning group was significantly positively correlated with two positive factors, PA and PE, as well as introverted and extroverted personality traits, and significantly negatively correlated with neuroticism and psychoticism. As shown in Figure 7 below, the higher the monthly income, the lower the financial pressure, the better the health status, and the better the living conditions, the higher the subjective well-being of the elderly was.

**Table 7:** Correlation analysis between personality factors and subjective well-being score of the elderly.

**Factor**	**Subjective well-being indicators**				
	**PA**	**NA**	**PE**	**NE**	**Total score**
EPQ-P	−0.169^**^	0.248^**^	−0.162^**^	0.252^**^	−0.258^**^
EPQ-E	0.223^**^	−0.085	0.235^**^	−0.031^**^	0.191^**^
EPQ-N	−0.232^**^	0.424^**^	−0.241^**^	0.434^**^	−0.412^**^

** *means the difference is very significant (p < 0.01)*.

Finally, stepwise regression analysis was conducted on multiple stratification of factors affecting subjective well-being, as shown in Table 8. It can be inferred that piano learning time, living conditions, health condition, neuroticism, psychoticism, and intra and extra characters had positive effects on the subjective well-being of the elderly.

**Table 8:** Multiple stratified stepwise regression analysis of subjective well-being factors.

**Variable**		**Standard regression coefficient β**	***R*^**2**^ value after adjustment**	***T*-value**	***P***
First level	Piano study time	0.134	–	3.347	0.001
	Living condition	0.146	0.134	3.465	0.001
	Health condition	1.197	–	4.664	0.000
Second level	Nervousness	−0.348	0.276	−8.421	0.000
	Psychoticism	−0.170	0.307	−4.239	0.000
	Intra and extra characters	0.111	0.312	2.784	0.006

## The Integration and Development of Piano Art and Media Education in the New Media Era Play a Role in the Long-term Care and Happiness of the Elderly People

### The Mechanism of Piano Art Integration and Development on Long-Term Care and Happiness of Elderly People

The physiological and psychological mechanism of subjective well-being produced by long-term piano art learning based on educational psychology was researched. In terms of physiological mechanism, happiness is transmitted from the brain. And a number of studies have shown that while playing the piano, enjoying the pleasure of the music agent stimulates the body to secrete a hormone to maintain the happy mood. In addition, studies have shown that the elderly who learn piano have positive personality, and dopamine can be produced in the process of learning to make the elderly feel happier, pleasant and upward (Wattanasoei et al., 2017). In addition, the elderly need to coordinate various body structures, such as eyes, hands, feet and trunk, in the process of learning piano. In the process of learning, various abilities in various aspects of physiology are developed. The visual spectrum does not just stimulate logical thinking in the older brain. At the same time, the visual performance of several lines of music in different parts of the elderly improves the cognitive processing speed of visual stimuli, and strengthens their training. While playing the piano, the movement of the fingers increases the area of the motor cortex and the area of the scalp that receives the signals, thus promoting blood circulation through the body. Finger movement effectively stimulates brain activity and brain blood, and brain aging has a significant delay. The movement of fingers and the use of stepping on the piano pedal, as well as the body rhythm in the performance process, enhance the flexibility and coordination of body movement, and improve the sense of movement and good touch of the elderly. As activity increases, so does the brain's processing power.

**Figure 8:**
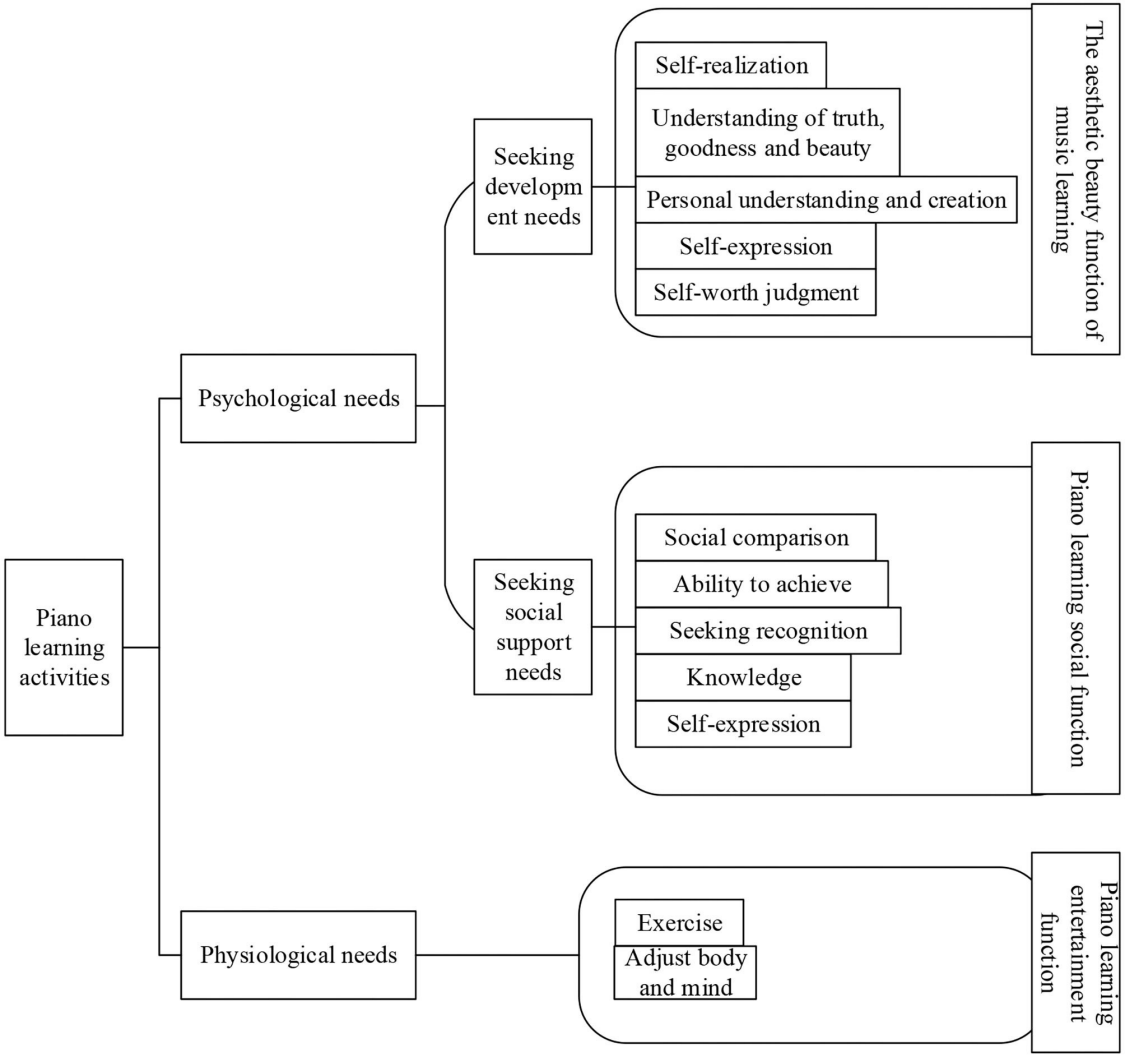
The model of psychological experience of piano learning to improve the subjective well-being of the elderly.

According to the active activity theory of social psychology of the elderly, social activity is the basis of individual activities, and maintaining contact with the society is a positive manifestation of adapting to aging. People also need activities in their old age. They should keep a good attitude and carry out all kinds of activities as before. And a positive self-image, sense of accomplishment and happiness are obtained in the activities. It is concluded that the goal of piano art learning activities for the elderly is to seek various social support and personal development needs. The happiness obtained by the elderly through music learning is also based on the needs of the elderly at all levels. The study of music by the elderly is considered as a psychological experience process to meet the needs of material life and self-worth. Figure 8 is a model of the psychological experience process of subjective well-being of the elderly in piano art learning based on educational psychology. It can be inferred that the subjective well-being of the elderly comes from both physical and psychological needs. Physical needs are met through exercise and physical maintenance, while psychological needs come from social support and personal development. Social support mainly includes social comparison, ability to achieve, seeking approval, knowledge, and self-expression. Self-development mainly includes self-consciousness, understanding of truth, goodness, and beauty, personal thinking ability and creativity, and self-value judgment.

### The Overall Evaluation of the Development of Piano Art and Media Education Integration on the Long-Term Care and Well-Being of the Elderly

**Figure 9:**
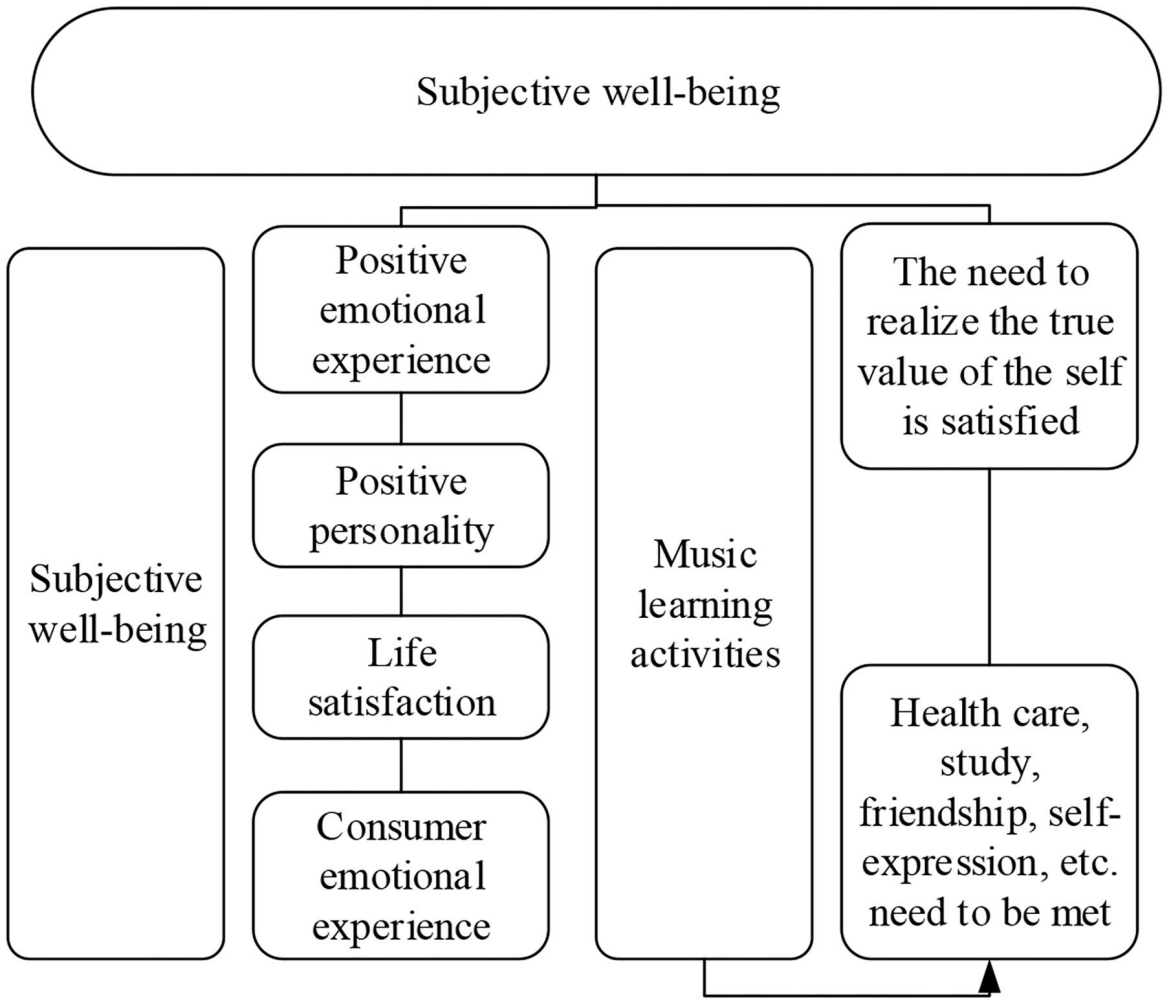
Path of piano learning influencing the positive experience of subjective well-being of the elderly.

The influence and mechanism of the integration and development of piano art and media education on the long-term care and happiness of the elderly in the era of new media were analyzed, and the path to the positive experience of subjective well-being of the elderly was put forward, as shown in Figure 9. In the long-term learning process of piano art, the elderly seems to have spiritual sustenance. Every day they have something to look forward to. No matter the study of difficulties, or the experience of piano art to obtain satisfaction, they are all a kind of transcendence. They acquire a positive personality. They have invested a great deal of passion and courage in relearning and practicing, and the psychological process is one of satisfaction and happiness. This further increases their life satisfaction.

In the age of new media, through the analysis of factors affecting the long-term care of the elderly, family economic status, living conditions, quality of life and health status are the main factors that affect whether the elderly get a good sense of well-being in long-term care. Good economic status, income, living conditions, and good health are the prerequisites for the happiness of the elderly.

## Conclusion

In the study, based on educational psychology, several scales of self-compiled personal information, the Ackerson personality inventory, and the memorial university of Newfoundland happiness scale were introduced for statement. The questionnaire method was adopted for information collection. Besides, the mechanism of the integration of piano art based on educational psychology and media on the long-term care and happiness of the elderly in aging society was summarized. According to the analysis, among the factors that affect the long-term care of the elderly, it is concluded that economic pressure, living conditions and health status are the main factors that affect the happiness of the elderly. Good economic pressure. Living conditions and health status have a positive effect on the well-being of the elderly. In the subjective happiness of the elderly, both PE and PA increase with the increase of piano learning time, while NE and NA decrease with the increase of piano learning time, indicating that piano art learning based on educational psychology can improve the happiness of the elderly. With the development of modern science and technology, in the age of new media, the elderly has more and more access to piano media, so it has a positive effect on the well-being of the elderly.

The study based on educational psychology provides certain guidance for the improvement of the life of the elderly in the present aging society. However, as piano art learning is a long-term accumulation process, there is no good quantitative standard. The relationship between the piano art learning behavior of the elderly and the relationship between long-term care and happiness are not discussed in the article. It is hoped that the follow-up research can be further explored.

## Data Availability Statement

The raw data supporting the conclusions of this article will be made available by the authors, without undue reservation.

## Ethics Statement

The studies involving human participants were reviewed and approved by Hunan Normal University Ethics Committee. The patients/participants provided their written informed consent to participate in this study. Written informed consent was obtained from the individual(s) for the publication of any potentially identifiable images or data included in this article.

## Author Contributions

All authors listed have made a substantial, direct and intellectual contribution to the work, and approved it for publication.

## Conflict of Interest

The authors declare that the research was conducted in the absence of any commercial or financial relationships that could be construed as a potential conflict of interest.
